# The Field of Science

**Published:** 1894-02-10

**Authors:** 


### The Field of Science.

On February 16th Professor E. Haeckel celebrates his sixtieth birthday, and it is proposed to do honour to the event. A marble bust of the great scientist will be placed in the Zoological Institute of Jena.

It will be a move in the right direction if lucifer match factories are compelled to keep a register of those persons who are employed in the works. So many distressing stories have been afloat from time to time regarding the results accruing physically on those employed in this particular industry, that certain stringent regulations were enforced some time ago throughout the factories of the United Kingdom. But a Special Commission has since been instituted, the members of which were appointed from the Chemical Works Committee, and certain new regulations will assuredly be enacted contingent on the recommendations of this particular body. To supplement already existing laws, for instance, it is suggested that periodical medical examination of match factory employés shall be enforced, and that the ventilation of the buildings shall be stringently attended to. The Committee lay stress on the fact that actual danger must exist for all workers falling victims to necrosis of the jaw in this industry where white and yellow phosphorus are used. Both Houses of Parliament have been presented with the report on the *modus operandi* of British factories, which the Commission has recently inquired into, and we have confidence that the additional precautions suggested thereby will prove a blessing to those engaged in the various manufactories.

The experiments made the other day at Argenteuil with the new explosive, Schnebelin, will be of great interest internationally. It strikes one as somewhat curious that this particular invention should originate primarily with a priest, after whom the new explosive takes its name. Numerous advantages are claimed for this powder by Abbe Schnebelin and his brother, Lieutenant Georges Schnebelin, who also is associated in the invention. By them it is asserted to be at least 20 per cent. more powerful than dynamite, which explosive has hitherto had no rival in this capacity. Besides the additional power motives, of economy will urge its acceptance, as Schnebelin has the further recommendation of being about 50 per cent. less expensive than any commodity of its kind. Added to this, the new powder is almost smokeless, gives little recoil, neither injures the gun, nor oxidises the barrel. The actual tests to which this has been submitted are varied and numerous. That Schnebelin has absolute insensibility to percussion has been successfully demonstrated, as also a remarkable resistence to heat under 540 Fahrenheit. More curious still, perhaps, is its imperviousness to wet; that is to say, that the powder after being wetted was proved not to have deteriorated at all in the operation, and was used in further experiments. In the application of Schnebelin to mining purposes the gases generated through the explosion are affirmed by the inventors to be quite harmless, and consequently thereon work may be resumed by those engaged in blasting at a quicker rate than is the case when other mediums are applied for the purpose.

An American contemporary computes that there are about seven hundred women doctors at the present time practising in Russia. Many of these occupy important positions in hospitals and workhouses, in educational establishments, in factories and works of various kinds, and in Government institutions, whilst others hold appointments in the service of municipal bodies. The remuneration for those different posts varies from about £200 downwards. So far as private practice is concerned, there is one woman doctor who makes an income of £1,800 per annum—a phenomenally good record. But the average income of the woman medical practitioner in private practice is something under £300 a year.

Of extreme interest to the scientific public is the appearance in the *Comptes Rendus* of the publication of Dr. M. N. Gamaleia's experiments on the artificial culture of cholera bacilli. In this short paper on the subject (which we much wish had assumed a more exhaustive form), the doctor tells us hcw he was able to increase the virulence of the cholera organism through cultivating it in media containing from 3, 4, up to 5 per cent. of ordinary salt. Nor were these results confined to any particular cultivation of the cholera bacillus, but were derived with cholera cultures obtained from various sources. Further on, Dr. Gamaleia tells us that when subsequently he inoculated these salt cultures into some pigeons and guinea pigs septicæmia developed in each case. It was found that one drop of the blood of animals thus infected, transmitted the disease upon re-inoculation into other bodies. These observations, comments *Nature*, support the theory that the unusual saline properties of the Elbe may have assisted in supplying the conditions which so greatly favoured the vitality and virulence of the cholera bacillus during the great epidemic at Hamburg.

The application of bacteriology to diseases of the eye is attracting considerable attention in the East and in Europe. We have it on American authority that 25 to 40 per cent. of the cases of blindness in that country are due to the malignant action of a minute microbe called by oculists a coccus. This microbe, which is sometimes described as a plant, sometimes as an insect, often appears on the conjunctiva of the eye a few days after the birth of an infant. As we all know regarding this, if it is resolutely attacked when it first makes its appearance it is extirpated without difficulty, and a patient who, but for medical treatment would have become hopelessly blind, retains his sight. By a recent law in New York State all midwives are required, under severe penalties, to report each instance which comes under their notice of inflamed eyes or red eruption over the eyelids of new-born children. Such cases are immediately treated by physicians in the employ of the Board of Health. Besides thus checking the contagion, this timely measure has been proved to have its socially economic side, since preserving the eyesight of the poor at the onset of disease, is a less costly measure than supporting them afterwards in a State asylum for the blind.

